# Quality of life and problems associated with obturators of patients with maxillectomies

**DOI:** 10.1186/s13005-017-0160-2

**Published:** 2018-01-05

**Authors:** Marwa Mohammed Ali, Nadia Khalifa, Mohammed Nasser Alhajj

**Affiliations:** 1 Department of Oral Rehabilitation, Faculty of Dentistry, University of Khartoum, Khartoum, Sudan; 2 Department of Preventive and Restorative Dentistry, Faculty of Dental Medicine, University of Sharjah, Sharjah, United Arab Emirates; 3 Department of Prosthodontics, Faculty of Dentistry, Thamar University, Dhamar, Yemen

**Keywords:** Quality of life, Obturators, Maxillectomy, Obturator functioning scale

## Abstract

**Background:**

Maxillary defects predispose patients to different undesirable effects. The aim of this study was to assess the quality of life (QoL) of patients with maxillary defects (acquired/congenital) wearing obturators.

**Methods:**

The study comprised 30 patients aged between 16 and 78 years. Interviews were conducted to collect information pertaining to patients; sociodemographic, self-reported function of obturator using Obturator Functioning Scale (OFS), self-evaluation of general health using Visual Analogue Scale (VAS), radiotherapy treatment, salivary gland removal, reconstructive surgery, neck dissection and length of time obturators were worn. Clinical examination included type of maxillectomy, Aramany classification of the defect, and evaluation of obturator function using the Kapur retention and stability scoring system.

**Result:**

Quality of life was affected significantly by marital status (*P* = 0.026). Married patients had better quality of life 61.3%, followed by divorced patients 38.8%, widowed 37.3% and the least QoL was detected in single patients 36.5%. Significant association between the type of maxillectomy and QoL was detected (*P* = 0.002). Retention of obturator prosthesis had a highly significant association with QoL (*P* < 0.001). Type of maxillectomy had a significant relation with obturator retention (*P* = 0.005). Stability had a significant correlation with QoL (*P* = 0.022). Obturator wearers who were treated with radiotherapy had lower QoL than those who were not treated with radiotherapy.

**Conclusion:**

Rehabilitation of patients with maxillary defects using obturator prosthesis is an appropriate and not invasive treatment modality. Results support that good obturators contribute to a better life quality.

## Background

One of the most important structures in the midface is the maxilla, which separates the oral, antral, and orbital cavities, and provides support to the eyeballs, lower eyelids, cheeks, lips, and nose. Furthermore, the maxilla plays a critical role in speech, swallowing, and mastication. Therefore, reconstruction of maxillectomy defects are particularly challenging for head and neck reconstructive surgeons [1]. Probably the most common of all intraoral defects are in the maxilla, and can be divided into defects resulting from congenital malformations and acquired defects resulting from surgery to remove oral neoplasms. Post-surgical maxillary defects predispose the patient to hypernasal speech, leakage of fluid into the nasal cavity, and impaired masticatory function [2].

The most frequent treatment modality for patients diagnosed with a maxillary malignancy is surgical removal of the tumour. This very often leaves an oronasal and/or oroantral defect, resulting in severe functional problems concerning mastication, deglutition, and speech. Therefore, an appropriate substitute for the tissue lost is inevitably necessary to restore function and regain quality of life (QoL) [3, 4]. Maxillofacial defects are usually complex, involving skin, bone, muscle, cartilage, and multiple layers of mucosa, so reconstruction of such defects is often challenging. A multidisciplinary approach is needed to rehabilitate such patients [5]. The maxillofacial prosthodontist has two primary objectives in the total rehabilitation of the maxillectomy patient, i.e., to restore the functions of mastication, deglutition, and speech and to achieve a normal orofacial appearance [6–8].

The benefit of prosthodontic rehabilitation of maxillectomy over autogenous tissue reconstruction is that it simplifies oncological surveillance [1]. The surgical site can be easily examined after removing the obturator prosthesis and tumour recurrence may be detected at that time [9]. A prosthesis used to close a palatal defect in a dentate or edentulous mouth is called an obturator [10] (from the Latin word *obturare*, meaning “to close up”) and is a disc or plate, natural or artificial, that closes an opening or defect of the maxilla as a result of a cleft palate or partial or total removal of the maxilla because of a tumour [8]. According to the Glossary of Prosthodontics Terms, an obturator is a prosthesis used to close a congenital or an acquired tissue opening, primarily of the hard palate and/or contiguous alveolar structures [11].

Individuals who require a maxillectomy often ask about the QoL they should expect following surgery. A well-constructed obturator can have a positive effect on individual’s QoL [12–15]. It is important for patients to be able to return to a normal life after maxillectomy without functional impairment or psychological trauma.

There is a lack of information pertaining to the relationship between subjective and objective assessment methods among patients with maxillectomy who are rehabilitated with obturator prostheses. The aim of this study was to evaluate subjectively functions such as mastication, swallowing, and speech along with aesthetics and psychological status in patients with maxillary obturators and to assess objectively the retention and stability of obturators. The subjective and objective assessment methods were then compared.

## Methods

This cross-sectional study was conducted at the Prosthodontic Department, Faculty of Dentistry, University of Khartoum, Sudan. The study population comprised patients with a maxillary defect who attended the Prosthodontic Department between April 2010 and October 2014. Patients were enrolled consecutively using the following inclusion criteria: adult of either sex, a maxillary defect, and wearing of an interim or definitive obturator for at least 1 month. The exclusion criteria were: recurrent disease and physical or mental instability. The patients were interviewed by the principal investigator (M.M.A.) and the collected data were entered in a spread sheet. The data collection sheet consisted of four parts as follows.

### Section A: sociodemographic information

Sociodemographic information included age, sex, marital status, level of education, and employment status. Patients were also asked about how long they have been wearing an obturator and whether they had received radiotherapy. Individuals with a new removable prosthesis were evaluated 1 month later to allow for the stimulatory effect on the oral cavity (foreign body) to subside [16].

### Section B: obturator functioning scale

The Obturator Functioning Scale (OFS) was developed at Memorial Sloan Kettering Cancer Center (New York, NY, USA) as a means of assessing self-reported functioning of an obturator. It was designed by Kornblith et al. [14] to assess eating ability, speech, and cosmetic satisfaction. To rate the items with higher scores (reflecting greater difficulty with obturator function), a 5-point Likert scale was used (“not at all”, “a little difficult”, “somewhat difficult”, “very difficult”, “extremely difficult”). One item, i.e., “difficulty talking on the phone”, was added to the scale to assess communication difficulties in the absence of visual cues [14]. For analysis, the responses were coded from 1 (“not at all”) to 5 (“extremely difficult”). The total score was then calculated by summing the responses “not at all”/“a little difficult”, and responses of “very difficult”/“extremely difficult” reflecting the mean score of the scale’s items, with higher scores reflecting greater difficulty.

An Arabic version of the OFS (OFS-Ar) was developed and adapted according to the translation guidelines using a forward-backward approach as follows. First, for the forward translation, an English version was translated by two separate teams of bilingual doctors. Both teams then worked together to create one combined translation. Second, for the backward translation, two independent bilingual translators produced two separate backward translations from the combined forward translation. Neither of the translators looked at the original English version of the questionnaire. A final version of the questionnaire was then produced by a team consisting of two Arabic linguistic experts and forward translators who revised all the translations and merged them into a final Arabic version [17]. The questionnaire was then pilot-tested on five patients. To check for the accuracy of the Arabic translation, each participant was interviewed after completing the questionnaire to make sure that the meaning of each part of it was clear and understandable. The typical question format was as follows: “Have you had (impact item) because of problems with your maxillary obturator?”

### Section C: visual analogue scale

This is a vertically graded scale that is numbered every 10 mm. The scale is similar to that on a thermometer and helps people to describe how well or bad they feel their health status to be. The subjects are instructed to put a mark on the scale that best reflected the intensity of their symptoms. The results are expressed in millimeters from zero (worst possible symptom severity) to 100 (no symptoms) [18–20].

### Section D: clinical examination

The clinical examination included recording type of maxillectomy, Aramany classification of the defect, presence of reconstructive surgery, surgical removal of salivary gland, and neck dissection. Obturator retention and stability were evaluated using the scoring system described by Kapur [21, 22].

### Statistical analysis

The data were analysed using descriptive statistics in terms of frequency distributions, means, and the standard deviation. The contingency coefficient was used for the association between selected variables and the retention and stability of obturators. Pearson coefficient was applied to test for a correlation between the OFS-Ar and the VAS. The independent-samples *t*-test (two groups) and analysis of variance (more than two groups) were used to compare QoL according to patient characteristics. A *P*-value <0.05 considered to be statistically significant. The Statistical Package For the Social Sciences version 17.0 (SPSS Inc., Chicago, IL, USA) and Microsoft Excel 2007 (Microsoft, Bothell, WA, USA) were used to enter and analyze the data.

The study protocol was approved in writing by the Ethical Committee of the University of Khartoum (Faculty of Dentistry). Written informed consent was obtained from all study participants after they had received a detailed explanation of the aims of the study.

### Pilot study

Five patients were investigated in a pilot study. These patients had maxillary obturators fabricated by MSc students under the supervision of prosthodontic specialists in the prosthodontic clinics. This preliminary study was performed to help assess the intelligibility of the questionnaire and the scales used, the feasibility of clinical evaluation of obturators, and construction of dummy tables.

## Results

### Section A: sociodemographic data

The sample consisted of an equal number of male (*n* = 15) and female (*n* = 15) patients. The mean patient age was 45.10 ± 19.03 years with a minimum age of 16 years. For statistical analysis, the variable of age was divided into groups as shown in Table 1. The majority of patients were in the age group of 40–59 years and the smallest number of patients were in the age group of 60–69 years.

**Table 1:** Characteristics of the participating patients

Variable		Percentage
Gender	Male	50.0
	Female	50.0
Age of patients	<20	13.3
	20–39	20.0
	40–59	40.0
	60–69	6.7
	≥70	20.0
Marital status	Single	30.0
	Married	53.3
	Divorced	13.3
	Widow	3.3
Education	Preschool education	30.0
	Primary school	30.0
	Secondary school	23.3
	University and above	16.7
Occupation	Professional	13.3
	Private business	13.3
	Labor	13.3
	Not employed	13.3
	housewives	33.3
	Retired	13.3
Radiotherapy treatment	Yes	26.7
	No	73.3
Duration of obturator wearing	Mean ± SD	24.47 ± 69.65

Fifty-three percent of the patients were married, 30% were single, 13.3% were divorced, and 3.3% were widowed. Thirty percent of respondents were not educated or had only received preschool education (‘khalwa’ or kindergarten), another 30% of respondents had completed primary school, 23.3% had completed secondary school, and 16.7% were educated at college, university, and postgraduate qualifications. Most of the patients were housewives (33.3%) and other occupation groups were equal in percentage (13.3%). The mean time that an obturator had been worn was 24.47 months (median 9.00 months, standard deviation 69.65 months, minimum 1 month, and a maximum of 384 months). Twenty-six percent of patients had received radiotherapy.

### Section B: obturator functioning scale

As shown in Table 2, 50% of subjects reported no or little difficulty chewing. Similarly, 50% had no or little leakage when swallowing food. The majority (80%) of respondents reported not having any difficulty with their voice before surgery. This is in contrast to after surgery, when four (13.3%) respondents reported extreme difficulty talking in public (although 70% reported no or little difficulty). More than 75% felt they had no or little nasal speech. Pronunciation of words was not a problem for more than 85% of respondents. About 86.2% (26 respondents) felt that their speech was not or a little difficult to understand, and 10% (three respondents) reported extremely difficulty when talking on the telephone. Dry mouth was reported as absent or slight by 66.7% (20 respondents) and two (6.7%) reported severe dryness. Four respondents (13.3%) were extremely dissatisfied with their appearance but more than 75% were not or a little dissatisfied. The clasps on the anterior teeth were reported to be extremely noticeable by only one patient (3.3%) while more than 75% reported that these clasps were not or a little noticeable. Seventy percent of respondents no numbness and two reported feeling numbness all the time. Avoidance of family or social events was not reported by 43.3% of subjects, but 20% reported a little avoidance and 13.3% reported avoidance all the time. Insertion and removal of the obturator was not or a little difficult for more than 80% of respondents but was extremely difficult for three (10%). More than half of the respondents (56.7%) felt that their upper lip “looked funny” and only five (16.7%) had no concerns about the appearance of their upper lip.

**Table 2:** Subjects’ responses to the Arabic version of Functional Obturator Scale

Variables		Not at all	A little	Somewhat	Very much	Extremely
Difficulty in chewing foods	Count	13	2	4	3	8
	%	43.3	6.7	13.3	10.0	26.7
Leakage when swallowing foods	Count	10	5	5	3	7
	%	33.3	16.7	16.7	10.0	23.3
Voice different from before surgery	Count	18	6	2	2	2
	%	60.0	20.0	6.7	6.7	6.7
Difficulty talking in public	Count	13	8	4	1	4
	%	43.3	26.7	13.3	3.3	13.3
Speech is nasal	Count	18	5	2	2	3
	%	60.0	16.7	6.7	6.7	10.0
Difficulty pronouncing words	Count	22	4	2	1	1
	%	73.3	13.3	6.7	3.3	3.3
Speech is difficult to understand	Count	22	4	1	0	3
	%	72.4	13.8	3.4	.0	10.3
Difficulty talking on the phone	Count	21	6	0	0	3
	%	70.0	20.0	0	.0	10.0
Mouth feels dry	Count	15	5	1	7	2
	%	50.0	16.7	3.3	23.3	6.7
Dissatisfaction with looks	Count	17	6	1	2	4
	%	56.7	20.0	3.3	6.7	13.3
Clasp on front teeth noticeable	Count	16	7	2	4	1
	%	53.3	23.3	6.7	13.3	3.3
Any area feels numb	Count	21	2	0	5	2
	%	70.0	6.7	0	16.7	6.7
Avoidance of family or social events	Count	13	6	3	4	4
	%	43.3	20.0	10.0	13.3	13.3
Difficulty to insert or remove obturator	Count	23	2	2	0	3
	%	76.7	6.7	6.7	.0	10.0
Upper lip looks funny	Count	11	6	5	3	5
	%	36.7	20.0	16.7	10.0	16.7

### Section C: visual analogue scale

The mean VAS score for patient health status was 65.83 mm (standard deviation 23.57 mm, median 70.00 mm, minimum 0 mm, and maximum 100 mm).

### Section D: clinical examination

The majority of obturator wearers presented with an Aramany class II defect (40%) followed by class I (33%), class IV (16.7%), class VI (6.7%), and class III (3.3%). Of the patients with an obturator, 73.7% had had a partial maxillectomy, 20% had had a hemi-maxillectomy, and 6.7% had had a subtotal maxillectomy. Only 26% of respondents had received radiotherapy. None had undergone reconstructive or salivary gland surgery. Forty-three percent of obturators presented with good retention, 30% with minimum retention, 13.3% with moderate retention, and 13.3% with no retention. Forty-six percent of obturators were adequately stable, 26.7% had some stability, and 26.7% were not stable (Table 3).

**Table 3:** Clinical examination of the participating subjects

Variable		Percentage
Aramany classification	Class I	33
	Class II	40
	Class III	3.3
	Class IV	16.7
	Class VI	6.7
Type of Maxillectomy	Partial	73.3
	Subtotal	6.7
	Hemi-maxillectomy	20
Neck Dissection	Yes	6.7
	No	93.3
Presence of reconstructive surgery	No	100
	Yes	0
Previous surgery for Salivary Gland	No	100
	Yes	0
Retention	No retention	13.3
	Minimum retention	30
	Moderate retention	13.3
	Good retention	43.3
Stability	No stability	26.7
	some stability	26.7
	Sufficient stability	46.6

Chi-squared test for the association between type of maxillectomy and Aramany classification with retention and stability of the obturator revealed significant association between type of maxillectomy and retention of the obturator. However, the other associations were not significant (Table 4). The influence of patients’ characteristics on the quality of life is presented in Table 5. The effect was significant for type of maxillectomy, retention of the obturator, and presence of radiotherapy. No any other significant effect was found.

**Table 4:** Association between type of maxillectomy and Aramany classification with retention and stability

	Contingency Coefficient	*P*-value
Type of maxillectomy and retention	0.621	0.005
Type of maxillectomy and stability	0.430	0.146
Aramany classification and retention	0.352	0.979
Aramany classification and stability	0.439	0.519

**Table 5:** Characteristics of patients and their influence on quality of life

Item scale	N	Quality of life%	Test statistic	*P*- value
		Mean ± SD		
Gender				
Male	15	38.8 ± 12.03	*T* = 0.598	0.585
Female	15	42.2 ± 18.47		
Age (years)				
<20	4	35.44 ± 8.49	*F* = 1.420	0.256
20–39	6	38.44 ± 16.33		
40–59	12	39.67 ± 16.12		
60–69	2	47.33 ± 7.72		
70+	6	58.67 ± 49.02		
Marital status				
Single	9	36.5 ± 10.2	*F* = 3.620	0.026
Married	16	61.3 ± 10.9		
Widowed	1	37.3		
Divorced	4	38.8 ± 15.3		
Education				
No education/ Khalwa/ kindergarden	9	37.63 ± 11.75	*F* = 0.595	0.625
Primary	9	39.67 ± 16.12		
Secondary	7	41.87 ± 15.62		
University +	5	45.78 ± 18.99		
Occupation				
Employed	12	35.22 ± 12.33	*T* = 1.581	0.125
Non employed	18	44.07 ± 16.54		
Duration (months)				
1–5	10	36.17 ± 11.26	*F* = 0.362	0.781
6–11	7	39.80 ± 9.96		
12–17	8	41.87 ± 17.17		
18 and above	5	43.87 ± 20.95		
Aramany classification				
Class 1	10	37.60 ± 12.80	*F* = 1.272	0.307
Class 2	12	41.33 ± 11.22		
Class 3	1	26.67		
Class 4	5	51.73 ± 26.72		
Class 6	2	29.33 ± 1.89		
Type of maxillectomy				
Partial Maxillectomy	22	74.67 ± 26.39	*F* = 7.847	0.002
Hemimaxillectomy	6	40.22 ± 13.24		
Sub-total maxillectomy	2	37.52 ± 11.50		
Retention				
No retention	4	30.56 ± 7.93	*F* = 8.602	<0.001
Minimum retention	9	40.74 ± 8.95		
Moderate retention	4	53.0 ± 10.46		
Good retention	13	60.0 ± 24.02		
Stability				
No stability	8	32.57 ± 10.7	*F* = 4.426	0.022
Some stability	8	46.0 ± 10.8		
Sufficient stability	14	49.0 ± 20.28		
Neck Dissection				
Yes	2	40.14 ± 15.66	*T* = 0.513	0.612
No	28	46.0 ± 14.14		
Radiotherapy				
Yes	8	35.27 ± 11.09	*T* = 3.727	0.001
No	22	55.0 ± 16.98		

## Discussion

Even though a large number of studies have investigated QoL after treatment for cancer [23–26], only a few articles have focused on patients using an obturator prosthesis after maxillectomy [9]. The present study investigated QoL after rehabilitation with an obturator prosthesis in Sudanese patients who had undergone maxillectomy. The study was conducted at the Faculty of Dentistry and Khartoum Dental Teaching Hospital of the University of Khartoum because these facilities are considered to be the main providers of dental care for patients who have undergone maxillectomy in the country.

In this study, 30 individuals with maxillary defects (irrespective of cause) and wearing maxillary obturators were investigated to determine their QoL at least 1 month after insertion of the obturator [16]. The sample size in this study was small because maxillary cancer is a rare tumour with high mortality. In fact, the number of samples in our study is within the range of 10–42 patients in other studies investigating patients who have undergone maxillectomy [12, 14, 23, 27–29]. The fact that the proportions of male and female patients in this study were equal may in part be attributable to the small sample size. Arigbede et al. [30] reported a similar sex distribution in their study, whereas others have reported a female [12, 13] or male [9, 14] predominance.

The mean patient age was approximately 45 years. The majority of patients were aged 40–59 years, which is similar to the finding by Riaz et al. [9] and most respondents were married (53%), which corresponds to the observations of other researchers [9, 12–14]. Most patients were not educated or had just received primary school education, which again is similar to findings by Khalifa et al. [31], and necessitates the use of simple questionnaires or scales that can be understood by these patients and yield more valid results. About a third of the patients were housewives (33%) and the remaining occupational groups were represented in equal percentages. The median time that the obturators had been worn was more than 10 months, which corresponds to the findings of Riger et al. [32].

The OFS has been used in numerous investigations [9, 13, 14, 27, 32], allowing comparisons to be made more easily. Half of the respondents reported little or no problems with leakage and chewing difficulties were noticed in the group investigated in this study. This may be attributable to the fact that nearly three quarters of the patients had undergone partial maxillectomy. This is in agreement with Irish et al. [13]. Patients in the present study reported having little difficulty with speech intelligibility or manipulating the obturator (e.g., insertion, removing, and cleaning) which is consistent with the observations of Kornblith et al. and Irish et al. [13, 14] Again, most patients were satisfied with their appearance and reported few or no aesthetic problems, similar to the previous observations of Irish et al. [13]. Nearly a quarter of individuals reported extreme difficulty accepting their appearance and thought that their upper lip looked peculiar. Kornblith et al. reported a higher percentage of patients with aesthetic problems [14]. Thirty percent of respondents felt that their mouth was very dry, which is comparable with the report by Irish et al. [13], but higher than the percentages in other studies [9, 12, 14]. This may be attributable to the fact that nearly a quarter of respondents had received radiotherapy. It is worth mentioning that no patient in the present study underwent any form of reconstructive surgery. As reported by Irish et al. [13], most of our respondents did not complain of numbness. More than a quarter of the study population avoided family and social events, which was less than the figures reported by other authors [13, 14].

The importance of using both general and disease-specific QoL measures has been emphasized by several authors [13, 14, 27, 33] because they each contribute unique information about QoL and can help to validate each other. In the current study, the VAS was used as a general QoL measure. Most of the patients rated their general health status as relatively good. The majority of obturator wearers presented with an Armany class II defect, similar to the findings of Arigbede et al. but different from those of Kumar et al. [29, 30].

Obturator retention and stability were evaluated using the scoring system described by Kapur, which is simple, applicable, and does not need any special instruments. The results using the Kapur scoring system revealed that the majority of obturators had good retention and stability. No statistically significant correlation was found between results for the OFS and the VAS. This was unexpected and not in agreement with previous studies in which OFS usually correlated significantly with other QoL measurements [13, 14, 27, 32]. Again, the small sample size in the current study could have partly contributed to this finding. It must also be remembered that difficulties in acceptance of a maxillary obturator are complex, and other factors that were not included in the present study may have had an effect on QoL. Female patients rated their QoL better than male patients. This could be because women are more self-motivated and more likely to attend for review visits. These observations are similar to those of Riaz et al. and Depprich et al. [9, 12].

In the current study, younger patients presented with worse QoL scores when compared with the older age groups. These findings are in agreement with those of Kumar et al. [29]. This may be explained by better acceptance of age-related health problems by elderly individuals and might also explain why they experience less distress related to cancer than their younger counterparts, who feel that their life span has been shortened and their QoL impaired because of the disease [29]. Married respondents evaluated their QoL better than single and widowed respondents. This was not surprising, and corresponds to observations by other investigators who have reported that the presence of loved ones helped people with cancer to enjoy a good QoL [12, 13].

This study revealed that patients with the highest level of education rated their QoL better than those with minimal education. This could be because of better awareness and understanding of instructions, manipulation methods, and limitations of maxillary obturators. Some of the previous studies support this finding [9, 12], while others have reported that level of education was not related to QoL [13]. Our patients with an obturator who were employed rated their QoL much lower than those who were not employed. This is not surprising given that some authors have commented that socioeconomic advantages, valued activities, and interests helped people with cancer enjoy a better QoL [13]. As expected, the longer a patient has worn the obturator the better the QoL, which corresponds to findings by Rieger et al. [32]. Further, as anticipated, individuals who had had a smaller area of palate resected (less than one quarter) had more retentive and stable obturators as well as better QoL than those who had more than one quarter of their palate resected. Again this is similar to the results reported by other authors [14, 34].

As expected, the retention and stability of obturators and QoL was better in our dentulous and partially dentulous patients than in those who were edentulous. This is consistent with the findings of Komaya et al., who reported that the presence of teeth in the maxillary dentition and different types of defect configuration had a significant correlation with the masticatory function score [34]. However, our results are different from those of Irish et al., who reported finding no difference in QoL between dentulous and edentulous individuals [13]. It was not surprising to find that good obturator function correlated with better QoL, as many previous studies have reported the same finding [9, 13, 14, 23, 27]. In addition to the small sample size, the type of analysis tools used may have contributed to the lack of statistically significant results when we examined the relationships between the study variables.

Whilst the Aramany classification system of maxillectomy defects has been widely used by prosthodontists, it is not the most commonly used by surgeons. This because Aramany classification system provides classification after healing has occurred and after the loss of any opportunity of immediate surgical reconstruction [35, 36]. On the other side, surgeons’ perspective of classification depends on the surgical resection [37, 38]. Hence, several maxillectomy classification systems have been proposed but, till now, no consensus has been reached [35–39].

It is important to encourage surgeons to keep the resection site as small as possible, because this is associated with better retention and stability of an obturator, which in turn leads to better QoL for wearer. Although free microvascularized flaps or pedicled flaps can be used as surgical means to repair maxillofacial defects, these flaps might be not suitable for large resections or defects. Instead, maxillofacial prostheses can be used effectively to obturate these defects. Several advantages can be achieved with obturators such as: replacing teeth as well as soft and hard tissues, allowing approximately normal speaking and swallowing for the patient. In addition, it prevent fluids leakage and communication between nasal and oral cavities. Moreover, it enhances the facial appearance as it provides support for the face tissues. Another benefit of the obturator is that it permits clear vision and may be early detection of tumor recurrence [2, 40, 41]. It would be useful to conduct studies using larger sample sizes to investigate QoL in patients using maxillary obturators further and to determine why certain individuals (female, married, unemployed and educated) have better QoL than others.

## Conclusion

Within the limitations of the current study, the following conclusions could be drawn: different variables can affect the patient’s response to a QoL questionnaire; the Arabic version of the OFS seems to be a valid instrument and can be used effectively in Arabic-speaking patients; there is no association between defect classification and patient QoL; a good obturator contributes to better QoL; and rehabilitation of patients with maxillary defects using well-designed obturator prostheses can be an appropriate and non-invasive means of treatment.

## Abbreviations

Ar: Arabic
OFS: Obturator functioning scale
QoL: Quality of life
VAS: Visual analogue scale
